# Crisis Standards of Care Guidelines for the COVID-19 Pandemic: Fresno Resource Allocation Guide (FRAG)

**DOI:** 10.7759/cureus.19662

**Published:** 2021-11-17

**Authors:** Patrick Macmillan, John Frye, Thianchai Bunnalai, Krista Kaups

**Affiliations:** 1 Internal Medicine, University of California San Francisco, Fresno, USA; 2 Ethics, Community Regional Medical Center, Fresno, USA; 3 Pediatric Critical Care Medicine, University of California San Francisco, Fresno, USA; 4 Trauma Surgery, University of California San Francisco, Fresno, USA

**Keywords:** triage protocols, resource allocation, trauma critical care, palliative and supportive care, medicine-pediatrics, medical ethics and pandemic

## Abstract

The coronavirus disease 2019 (COVID-19) pandemic has forced healthcare providers and policymakers to look candidly at the possibility that critical care resources, such as ventilators, medical staff, extracorporeal membrane oxygenation (ECMO), bilevel positive airway pressure (BiPAP) machines, and high-flow oxygen, may become scarce or depleted if the virus continues to move throughout the United States unabated. With hospitalizations and ICU occupancy rates rapidly increasing all over the US, we must face the uncomfortable truth that a triage system, much like on the battlefields of war, will need to be implemented. Ethical concerns abound, but the process for addressing limited resources must continue to be explored.

Multiple frameworks have previously been developed to address the use of limited medical resources during catastrophic public health emergencies. Many crisis care guidelines and protocols address the maximizing of surge capabilities and allocation of resource use (specifically, ventilators). While overwhelming scenarios unfolded in Europe and then on the East Coast of the United States in March of 2020, our hospital system in central California was obligated to consider previously unimaginable scenarios. In an effort to pro-actively address these, an expert group, consisting of intensivists (adult and pediatric), trauma surgery, palliative care, and ethicists was organized to develop guidelines for resource allocation to be utilized for our medical system in the event of a public health emergency. As part of this process, existing guidelines and consensus documents were reviewed. A novel system for ventilator allocation was developed, termed the Fresno Resource Allocation Guide (FRAG). As the pandemic continued to surge into 2021, we began to look at other resources, such as oxygen delivery systems other than ventilators, as well as healthcare team members.

This resource allocation guide takes into account a depletion in critical care supplies for adults and children. It employs ethical principles and evidence-based tools for critical care.

## Introduction

Despite an abundance of vaccine availability in the United States, the COVID-19 pandemic continues to stretch the resolve of many in the healthcare field. Hospitalizations and intensive care unit (ICU) occupancy rates are increasing all over the US. Children are now becoming more frequently infected with severe acute respiratory syndrome coronavirus 2 (SARS-CoV-2) and requiring critical care resources. Hospitals in the Southern United States have had difficulties keeping adequate oxygen supplies as COVID-19 cases and hospitalizations continue. The upward trend of this curve is the unfortunate result of Americans who remain unvaccinated and the high infectivity rate of the Delta variant [1]. Realizing this strain, healthcare providers and hospital policymakers are evaluating the process for addressing limited resources.

As the coronavirus disease 2019 (COVID-19) pandemic initially spread across the globe, medical communities and healthcare policy agencies were compelled to consider and manage both actual and potential resource limitations such as mechanical ventilators, medical staff (i.e. nurses), and extracorporeal membrane oxygenation (ECMO) machines.

Multiple frameworks have previously been developed to address the use of limited medical resources during catastrophic public health emergencies [2]. Many crisis standards of care guidelines and protocols address the maximizing of surge capabilities and allocation of resource use (specifically ventilators) [3]. While overwhelming scenarios unfolded in Europe and then on the East Coast of the United States in March of 2020, our hospital system in Central California was obligated to consider previously unimaginable scenarios. In an effort to proactively address these, an expert group, consisting of intensivists (adult and pediatric), trauma surgeons, palliative care personnel, and ethicists, was organized to develop guidelines for resource allocation to be utilized for our medical system in the event of a public health emergency. As part of this process, existing guidelines and consensus documents, including Biddison et al. and the New York State Department of Health documents were reviewed [4-5]. After careful examination, a novel system for ventilator allocation was developed, termed the Fresno Resource Allocation Guide (FRAG). As the pandemic surged in California during the fall and winter months our team reconvened to address the surge capacity in our hospital. We saw that considering all critical care resources would be germane to the discussion, not only ventilators.

Our workgroup priorities focused on developing an ethical and evidence-based approach to patient evaluation and resource apportionment (ventilators initially but later evolved into critical care resources) in a situation where surge capacity was exceeded, either with patients with respiratory failure secondary to COVID-19 or in other cataclysmic situations. Among the goals for this crisis care guidelines were:

1. The incorporation of reproducible, objective criteria to evaluate patients to allow transparency in this process

2. The development of easily assessed criteria from readily available information

3. The utilization of accepted triage principles and mechanisms

The protocols and algorithms we recommend are a modified standard of care based on dramatically reduced critical care resources during a healthcare crisis. There existed a collective consensus among our COVID-19 task force and its constituents that there be defined support from administration and legal regarding the veracity of the triage officer and the respective committees overseeing the resource allocation process. We also relied on the counsel of multiple ethics specialists as we developed our resource allocation guide. Lastly, we collaborated with our pediatric intensivists and developed a scoring system that utilized the Pediatric Logistic Organ Dysfunction (PELOD) score [6].

## Technical report

Community medical centers in Central California include three acute care hospitals with 950 beds and 121 ICU beds. Fresno is a metropolitan area, with a population of approximately 650,000, located in the Central Valley of California. The Central Valley (also known as the San Joaquin Valley) is both ethnically diverse and economically challenged. The region is medically underserved, with fewer physicians per capita than most other metropolitan regions of the state [7]. Our lower vaccination rates compared to other areas in California may also indicate a mistrust for regional medical establishments. In consideration of the local factors, it was essential that the allocation scheme be as transparent as possible.

Multiple allocation schemes, noted in previous sections, have effectively described the identification and function of triage officers and teams in the implementation of resource allocation; this will not be reiterated in the present manuscript. While some triage protocols have focused solely on the patient’s current clinical status, most have incorporated measures of both the short-term and long-term likelihood of benefitting from scarce resources (most often mechanical ventilation). Our focus widened as the winter came to include critical care resources. In the field of palliative care, comorbid conditions are often weighed when goals of care are discussed. Additionally, data demonstrate that those infected with COVID-19, who also suffer from multiple comorbid conditions, have the worse outcomes [8].

Including an approach that addresses both acute and chronic medical conditions in the most evidence-based way seems to be the most ethically and morally defensible. Given our diverse community, which includes Latinx, Hmong, African-American, and Punjabi, we did not want our resource allocation policy to be interpreted as discriminatory or arbitrary. However, we medically minister to one of the most underserved areas in the country, where we see conditions that some hospitals around the US may not encounter.

The majority of published guidelines have utilized the Sequential Organ Failure Assessment (SOFA) score for the initial triage evaluation of adults (Table 1) [9].

**Table 1:** Sequential Organ Failure Assessment Sequential Organ Failure Assessment (SOFA); Glasgow Coma Scale (GCS); Mean Arterial Pressure (MAP); Partial Pressure of Oxygen (PaO2); Fraction of Inspired Oxygen (FiO2)

SYSTEM	SOFA	
Central Nervous System	0	GCS = 15
	1	GCS = 13-14
	2	GCS = 10-12
	3	GCS = 6-9
	4	GCS = < 6
Cardiovascular catecholamine doses are given as mcg/kg/min	0	MAP > 70 mmHg
	1	MAP < 70 mmHg
	2	Dopamine < 5 OR Dobutamine any dose
	3	Dopamine 5.1-15 OR Epinephrine < 0.1 OR Norepinephrine < 0.1
	4	Dopamine > 15 OR epinephrine > 0.1 OR norepinephrine > 0.1
Respiration PaO2/FIO2, mmHg	0	> 400 (53.3)
	1	< 400 (53.3)
	2	< 300 (40)
	3	< 200 (26.7) w/respiratory support
	4	< 100 (13.3) w/respiratory support
Renal Creatinine, mg/dL (µmol/L) urine output (UO) mL/d	0	< 1.2 (110)
	1	1.2-1.9 (110-170)
	2	2.0-3.4 (171-299)
	3	3.5-4.9 (300-440) UO < 500
	4	> 5.0 (440) UO < 200
Coagulation Platelets, x 10^3^/µL	0	> 150
	1	> 150
	2	< 100
	3	< 50
	4	< 20
Liver Bilirubin, mg/dL (µmol/L)	0	< 1.2 (20)
	1	1.2-1.9 (20-32)
	2	2.0-5.9 (33-101)
	3	6.0-11.9 (102-204)
	4	> 12 (204)

However, a number of measures for evaluating comorbid conditions have been used, ranging from an overall clinical impression, American Society of Anesthesiologists (ASA) scoring, to frailty index to assist in times of crisis when scarcity of resources may become a paramount concern [10-11]. In the present scheme, a modified Charlson Comorbidity Index (CCI) was incorporated in addition to the SOFA score to create a scoring method (Table 2) [12].

**Table 2:** Charlson Comorbidity Index Electrocardiogram (ECG); Centimeter (cm); Cardiovascular Attack (CVA); Transient Ischemic Attack (TIA); Human Immunodeficiency Virus (HIV)

SCORE	CONDITION
1	Myocardial Infarction (history, not ECG changes only)
	Congestive heart failure
	Peripheral vascular disease (includes aortic aneurysm ≥6cm)
	Cerebrovascular disease: CVA with mild or no residua or TIA
	Dementia
	Chronic pulmonary disease
	Connective tissue disease
	Peptic ulcer disease
	Mild liver disease (without portal hypertension, includes chronic hepatitis)
	Diabetes without end-organ damage (excludes diet-controlled alone)
2	Hemiplegia
	Moderate or severe renal disease
	Diabetes with end-organ damage (retinopathy, neuropathy, nephropathy, or brittle diabetes)
	Tumor without metastases (exclude if >5years from diagnosis)
	Leukemia (acute or chronic)
	Lymphoma
3	Moderate or severe liver disease
6	Metastatic solid tumor
	AIDS (not just HIV-positive)

For patients under 18 years of age, the PELOD score has been validated and widely used to assess the severity of multiple organ dysfunction in the pediatric intensive care unit. The lower the score, in both adults and children, the more likely a patient would benefit from intervention, including critical care and non-invasive ventilation (NIV) support. Our system utilizes triage officers and an appeals committee to ensure a degree of fairness and excludes treating physicians from making decisions regarding resource allocation. Physicians and families retain the right to appeal the decision but not the established process, which is similar to most resource allocation protocols.

Ethical considerations 

In addition to its clinical utility and practicability, a triage framework is shaped by its ethical defensibility. In a public health crisis, it is important to make sure that the benefits one provides to the health of the community do not compromise the procedural fairness of a process that attempts to care for all patients [13]. Procedural fairness is one component of justice, a foundational principle in both clinical ethics and public health ethics. Thus, any prioritization of one category of patients over another should only be made if (a) its goal is ethically appropriate, and (b) the method of achieving that goal is fair in its application.

We are not the first to propose using the CCI to help assign priority. Others have noted that it can provide for the goal of maximizing the most life-years patients will receive, which can serve as a tiebreaker should two patients have an equal chance of survival to discharge [14]. The goal of saving the most life-years is noted as a potential ethical value in a rationing situation, on the basis that, on balance, more years of life lived benefit both the individual living them and the community they participate in and contribute to. If society has to choose whether to give a scarce resource to person A (life expectancy: 10 years) or person B (life expectancy: 20 years), the life years approach says that all else equal, person B should get the resource because they will benefit more than person A, and society will benefit more from their longer life.

To our group, this goal seemed both ethically questionable as well as difficult to apply fairly. Using younger age as a tiebreaker would achieve the same goal in a more direct fashion. One might argue that the statistically significant differences in life expectancy across sex, race, income level, or location of residence imply that each variable could serve as a tiebreaker and show a marginal benefit in maximizing life-years. It is true that the ethics literature on triage considers the merit of using factors such as race or residence in an area of significant deprivation based on the Area Deprivation Index. However, the proposed frameworks incorporate such factors to help prioritize patients who may be disproportionately vulnerable to COVID-19 and its potential risks and burdens, rather than to prioritize those patients already having better health and access to health care. Similarly, patients with disabilities may have a shorter life expectancy than other patients (or so their physicians might judge). But to use lack of disability as a tiebreaker because of projected life-years saved would be inappropriate discrimination.

On a separate consideration, it was not clear to us why maximizing life-years was the proper province of triage frameworks in a pandemic [15]. We grant that when all else is equal, longer life for each patient is a worthy goal. However, it is inappropriate to game the system in order to maximize a statistical measure of population health by sacrificing those most at risk of shorter life so that the persons who are already healthier may receive more benefit. To do so would only exacerbate existing inequalities in healthcare access.

For all these reasons, FRAG does not attempt to maximize life-years. When considering how to best benefit our patients optimally and justly, each framework must determine the “horizon of benefits,” that is, the farthest into the future the framework considers a patient’s potential condition when determining priority. When responding urgently to a public health crisis, we felt it appropriate for the horizon of benefits to be a “relatively brief” time after discharge, in the order of weeks to months. This allowed us to consider more than mere survival to discharge but also whether a patient might have survived their current illness but be left in such a poor health state that death was likely to follow soon.

The modified Charlson Comorbidity Index has demonstrated utility in predicting mortality and severity in COVID-19 patients. In one small study, a resource allocation framework combining SOFA and CCI better correlated with 14-day outcomes than approaches using SOFA alone or adjusting for severe or major comorbidities. CCI’s use also reduced the need to rely on lottery tiebreakers, showing that it helped clinicians distinguish categories of patients in clinically meaningful ways. It should be noted that the CCI was not designed for use in triage, to help judge either short-term risks or long-term benefits. Its use also may have the foreseen but unintended effect of mimicking a life-year maximization approach. Nevertheless, we felt that the CCI was both clinically useful and ethically defensible within the horizon of benefits we considered.

The CCI may correlate with other variables, such as race, socioeconomic status, and disability. Its inclusion in our framework for medically and ethically defensible reasons does not eliminate the need to account for whether the patient is a member of a vulnerable population. While direct inclusion of a patient's vulnerability into the calculation of the triage score or a tiebreaking method might seem the ethically preferable or required way to respond to societal inequalities known to impact health and healthcare, the inclusion of any particular metric for vulnerability is only as good as the evidence supporting it; otherwise, it would amount to a well-intentioned and well-reasoned guess at what the disproportionate impact is and what a proportional remedy should be. Such evidence may be presented at the state, national, or international level, but it may also become evident in local allocation decisions if a triage framework is imposed for a significant amount of time. Rather than anticipate such evidence, FRAG relies on the principle of fairness, to treat people equally. An ongoing analysis of allocation decisions must also be performed to determine whether any vulnerable groups are disproportionately bearing the negative consequences of any triage framework. As evidence of the disproportionate impact of COVID-19 on different vulnerable populations continues to come to light, FRAG can be modified by individual hospitals to best ameliorate these effects, reflecting the impact experienced by their local communities.

FRAG promotes the importance of physicians ensuring that each patient gets an individualized assessment of their clinical condition. Such an assessment, coupled with a robust goal-of-care discussion with the patient or family, can promote both understanding and shared decision-making at a time when confusion, ignorance, and disagreement can harm patients and clinicians as much as a lack of resources. Proactive goal-of-care discussions sensitize the team to the patient’s condition and value and motivate agreement on realistic ways to actualize patient values given resource scarcity. Ongoing analysis of allocation decisions must also be performed to determine whether any vulnerable groups are disproportionately bearing the negative consequences of any triage framework.

Triage process

Once hospital leadership has determined that a crisis situation exists, the application of the resource allocation model is set in motion. The triage officer, which has been recruited and trained, will review a daily rounding list that our Epic IT personnel designed. Attending physicians may also trigger a request for the Triage Officer to evaluate patients - this may occur in patients that are not intubated but may soon need to be. Our hospital Command Center may also trigger a request to the Triage Officer for evaluation.

**Figure 1:**
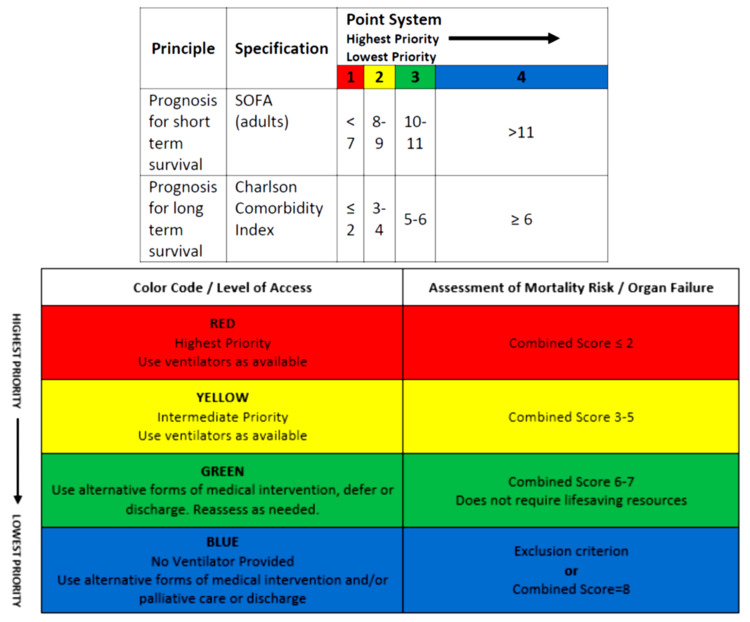
Adult Fresno Resource Allocation Guide Scoring System Sequential Organ Failure Assessment (SOFA)

Adult patients are subject to the following two scores (short-term survival assessment + long-term survival assessment) and are given a combined raw score. In the FRAG system, the SOFA score is first calculated using available clinical data with a maximal potential score of 24. The CCI is then evaluated and SOFA and CCI scores are then summed and a priority group determined. Per previous models, the groups are color-coded both to facilitate grouping and discussion. 

 i. Short-Term Survival: assessment of mortality risk using the SOFA score.

 ii. Long-Term Survival: assessment of prognosis using the Charlson Comorbidity Index (CCI).

 iii. The combined score determines the priority color for resource allocation (Figure 1).

 iv. Summary of prioritization:

§ · Red: highest priority, use ventilator as available. Reassess in 48 hours.

§ · Yellow: intermediate priority, use ventilator as available. Reassess in 48 hours.

§ · Green: use alternative forms of medical intervention, defer or discharge. Reassess as needed.

§ · Blue: no ventilator provided, use alternative forms of medical intervention and/or palliative care or discharge.

Pediatric patients have a process that is similar in scope to the adult framework.

 v. Assessment of severity of illness using the Pediatric Logistic Organ Dysfunction (PELOD) score (Table 3) [16].

**Table 3:** Pediatric Logistic Organ Dysfunction Score Not Applicable (NA); minute (min); micromol per Liter (Mmol/L); fraction of inspired oxygen (FiO2); partial pressure of carbon dioxide in arterial blood (PaCO2); partial pressure of oxygen in arterial blood (PaO2); millimeters of Mercury (mm Hg); kilopascal; (kPa); Liter (L); International unit per liter (IU/L); International normalized ratio (INR)

		Scoring system			
		0	1	10	20
Organ dysfunction and variable					
Neurological					
	Glasgow coma score	12–15	7–11	4–6	3
		and		or	
	Pupillary reactions	Both reactive	NA	Both fixed	NA
Cardiovascular					
	Heart rate (beats/min)				
	<12 years	≤195	NA	>195	NA
	≥12 years	≤150	NA	>150	NA
		and		or	
	Systolic blood pressure (mm Hg)				
	<1 month	>65	NA	35–65	<35
	1 month-1 year	>75	NA	35–75	<35
	1–12 years	>85	NA	45–85	<45
	≥12 years	>95	NA	55–95	<55
Renal					
	Creatinine (μmol/L)				
	<7 days	<140	NA	≥140	NA
	7 days−1 year^‡^	<55	NA	≥55	NA
	1–12 years^‡^	<100	NA	≥100	NA
	≥12 years	<140	NA	≥140	NA
Respiratory					
	PaO_2_ (kPa)/FIO_2_ ratio	>9·3	NA	≤9·3	NA
		and		or	
	PaCO_2_ (kPa)	≤11·7	NA	>11·7	NA
		and			
	Mechanical ventilation^§^	No ventilation	Ventilation	NA	NA
Haematological					
	White blood cell count (×10^9^/L)	≥4·5	1·5–4·4	<1·5	NA
		and		or	
	Platelets (×10^9^/L)	≥35	<35	NA	NA
Hepatic					
	Aspartate transaminase (IU/L)	<950	≥950	NA	NA
		and		or	
	Prothrombin time(or INR)	>60	≤60	NA	NA
		(<1·40)	(≥1·40)		

 iii. PELOD score determines the priority color for resource allocation (Figure 2).

**Figure 2:**
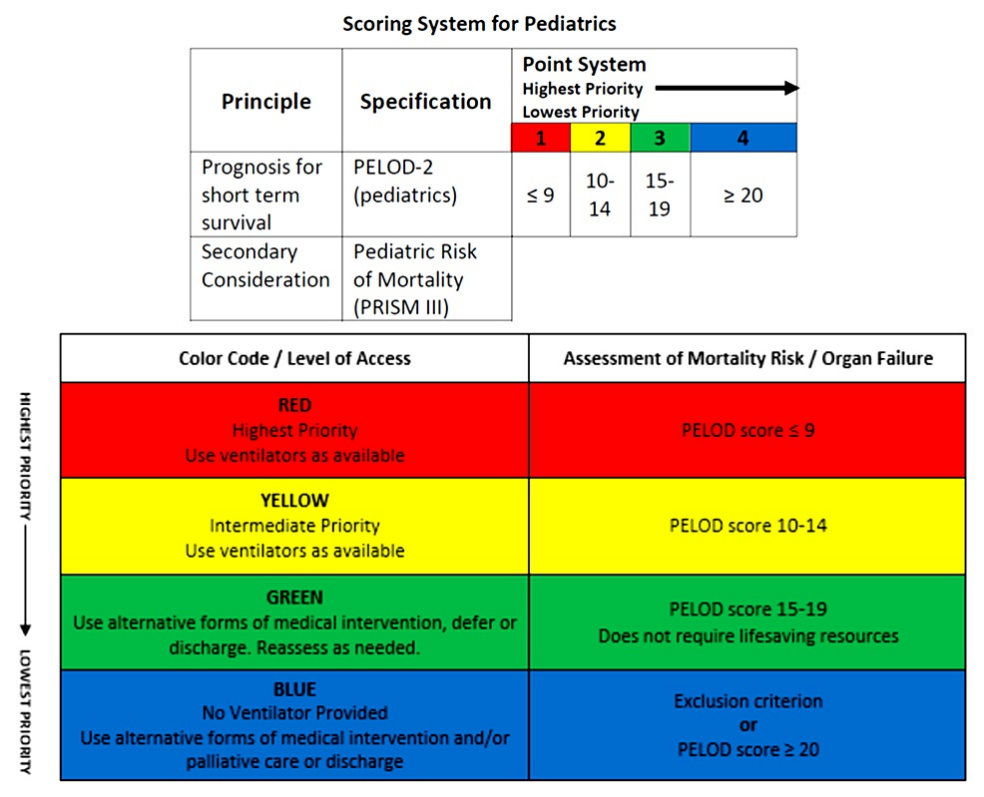
Pediatric Fresno Resource Allocation Guide Scoring System Pediatric Logistic Organ Dysfunction (PELOD-2) Pediatric Risk of Mortality (PRISM III) Calculator

 iv. Summary of prioritization:

 o · Red: highest priority, use ventilator as available. Reassess in 48 hours.

 o · Yellow: intermediate priority, use ventilator as available. Reassess in 48 hours.

 o · Green: use alternative forms of medical intervention, defer or discharge. Reassess as needed.

 o · Blue: no ventilator provided, use alternative forms of medical intervention and/or palliative care or discharge.

Exclusion criteria for adult patients 

i. Cardiac arrest, limited to unwitnessed arrest, recurrent arrest without hemodynamic stability, arrest unresponsive to standard interventions and measures, trauma-related arrest;

ii. Irreversible age-specific hypotension unresponsive to fluid resuscitation and vasopressor therapy;

iii. Traumatic brain injury deemed non-survivable;

iv. Severe burns: where predicted survival is ≤ 10% even with unlimited aggressive therapy, or;

v. Any other conditions resulting in immediate or near-immediate mortality, even with aggressive therapy.

Exclusion criteria for pediatric patients

Pediatric patients with conditions that result in immediate or near-immediate mortality even with aggressive therapy are excluded. Examples of underlying diseases that may predict poor short-term survival or long-term resource demand may include but are not limited to:

 I. Cardiac arrest not responsive to PALS interventions within 20 minutes of appropriate resuscitation;

 II. Recurrent cardiac arrest, without interval hemodynamic stability;

 III. Irreversible age-specific hypotension unresponsive to fluid resuscitation and vasopressor therapy;

 IV. Traumatic brain injury with no motor response to painful stimuli;

 V. Burn > 91% of BSA for children < 2 years of age;

 VI. Congenital heart disease with a poor chance of long-term survival;

 VII. Cardiomyopathy with ejection fraction < 25% and pulmonary edema unresponsive to therapy;

 VIII. Severe chronic lung disease, including pulmonary fibrosis, cystic fibrosis, obstructive or restrictive diseases requiring continuous home oxygen or mechanical ventilation use prior to the onset of acute illness;

 IX. Central nervous system, solid organ, or hematopoietic malignancy with poor prognosis for recovery;

 X. Liver disease with ascites, history of bleeding, fixed coagulopathy or encephalopathy; acute hepatic failure with hyperammonemia;

 XI. Acute and chronic and irreversible neurologic impairment, what makes the patient dependent for all personal care (e.g. severe stroke, congenital syndrome, persistent vegetative state, severe dementia, etc.);

In the event two (or more) patients are placed in a priority color and have the same score, their individual raw scores will be reevaluated and additional criteria may be applied, reducing the raw score by one. Criteria may include pregnant women and healthcare workers, which our COVID-19 group decided to utilize.

Patients on reassessment are moved into the appropriate category based on the most recent scoring. Should the scoring indicate that no critical care resources are to be provided, this will be communicated to the treating physician. For adult patients, the SOFA score is used for the reassessment process. The process still relies on sound clinical assessments. For pediatric patients, the PELOD score, in conjunction with secondary assessment measures, such as the Pediatric Risk of Mortality-III (PRISM) score, and multidisciplinary evaluation are used for the reassessment process.

## Discussion

The surge in COVID-19 cases around the country and in the Central Valley of California demands the adoption of a policy for scarce critical-care resource allocation. The CCI, used by itself, is a tool to predict long-term mortality. However, our rationale to use it alongside the SOFA score was not to predict long-term survivability outside the hospital but to look at how chronic life-limiting conditions blend with SARS-CoV2 and its other clinical manifestations. The CCI, a method of predicting mortality by classifying or weighting comorbid conditions, has been utilized by health researchers for years. Since the publication of the original Charlson et al. article in 1987 [12], the paper has been cited innumerable times and the index has been validated for its ability to predict mortality in various disease subgroups. These studies consistently demonstrate that the Charlson index is a valid prognostic indicator for mortality.

As the pandemic continued and more data became available, we saw that more males and those with a comorbidity such as hypertension, diabetes, cardiovascular disease, or chronic lung disease are dying from the disease at a higher rate. Cancer was also associated with a higher incidence of death. More recent data from the United States indicate cardiovascular disease, diabetes mellitus, and lung disease as the conditions most associated with death as a result of COVID-19 [17]. Dementia and kidney and liver disease are also noted to be associated with an increased rate of death. Analysis in Scotland showed that nine in 10 people who died with suspected or confirmed coronavirus infection had a pre-existing health condition [18].

Additionally, we were sensitive to the ethics around discrimination. The early cases we saw over the summer months and those analyzed suggest a preponderance of individuals with comorbid medical conditions have a disproportionately poorer hospital course or have complications far worse than those without them. The coronavirus infection itself creates unique healthcare outcome disparities for which the causes are not fully understood. Previous healthcare status and socioeconomic stressors certainly play a role in these disparate groups.

Typical goals of care conversations take into account debility, frailty, and multiple medical conditions as individuals and families grapple with emotional end-of-life discussions. An oncologist will consider a patient's functional status using the Eastern Cooperative Group (ECOG) scoring system before proceeding with chemotherapy, given the potentially debilitating side effects of treatment. Palliative Performance Scales gauge functional status at end of life and assist with determining prognosis [19]. All of these scores factor one’s ability to withstand further aggressive intervention, and multiple comorbid conditions such as dementia, heart failure, or emphysema all contribute to poor performance status. It seems counterintuitive to ignore such medical facts when looking at survivability in a scarce resource scenario. Therefore, we concluded that a system built on looking at acute and chronic medical conditions constituted a rational and defensible approach.

Our system does not mandate that individuals who come into the hospital be denied treatment for COVID-19 infection. Our allocation system strictly applies to a scenario in which a surge crisis exists, and we are depleted of critical care resources. In a non-surge/crisis situation, we advocate for a continued standard of care alongside palliative care assistance when warranted. Palliative care consult services in hospitals are critical in normal times but particularly during this pandemic when complex goals of care discussions are required. 

We have given credence to the value of clinical judgment as well. Our triage officer and team exist to track the number of patients we have that require critical care resources and compute the combined SOFA and CCI scores. Triage officers communicate with families using scripts disseminated by the Center for the Advancement of Palliative Care (CAPC) [20]. Physicians’ clinical assessments are paramount. Physicians or providers have the ability to appeal the decision of the triage team. We recognize that patients may appear sicker when only a chart review is done. Palliative care physicians and hospitalists often find common ground related to overall prognosis. The appeals process plays a vital role that allows for the sound clinical judgment of physicians to intervene when making critical distinctions for conditions such as heart failure that have varying degrees of severity.

Finally, a multitude of ethical questions and different perspectives have surfaced during our initial and ongoing task force discussions. We must keep in mind that we are in uncharted waters for our lifetime. The Influenza pandemic of 1918 certainly draws parallels, but technology is vastly different. Our resource allocation document has no authority in times other than a pandemic or a national healthcare crisis and only when critical care resources become scarce. As the pandemic crisis worsens, we are finding that other resources besides ventilators, such as ICU beds, medical staff, and BIPAP machines (critical care resources), are in short supply.

It should be made clear that our decision-making strategy for this resource allocation or crisis care guide should not be construed by healthcare providers or the public as a mechanism for abrogating care for severely ill patients. When aggressive life-sustaining care is deemed medically non-beneficial by our scoring system and triage team or treating providers, we have in place a mechanism to provide intensive compassionate care that includes physical, emotional, and spiritual symptom management.

## Conclusions

The COVID-19 pandemic has exposed many gaps in our healthcare system, including the scarcity of vital life-saving medical equipment and critical care resources. Our scoring system considers acute and chronic medical conditions in an evidence-based and ethically balanced fashion as it relates to survivability in the hospital from COVID-19 respiratory failure and other sequelae.
